# Therapeutic factors that promote recovery in high-risk adolescents intensive group psychotherapeutic MBT programme

**DOI:** 10.1186/s13034-019-0263-6

**Published:** 2019-01-10

**Authors:** Kirsten Hauber, Albert E. Boon, Robert Vermeiren

**Affiliations:** 1 De Jutters, Centre for Youth Mental Healthcare Haaglanden, Dr. Van Welylaan 2, 2566 ER The Hague, The Netherlands; 2 Lucertis, Child and Adolescent Psychiatry, Rotterdam, The Netherlands; 3 Department of Child and Adolescent Psychiatry, Curium-Leiden University Medical Centre, Leiden, The Netherlands

**Keywords:** Ego narratives, Group therapy, Adolescents, MBT

## Abstract

**Background:**

The aim of this study was to investigate whether therapeutic factors as identified by Yalom and potential additional therapeutic factors could be found in the qualitative individual reports of high-risk adolescents with personality disorders at the end of an intensive group psychotherapeutic MBT programme and whether the therapeutic factors were related to therapy outcomes.

**Methods:**

At the end of treatment, 70 adolescents were asked to write a farewell letter. Content analysis of the letters was performed by two independent raters, using the 12 therapeutic factors of Yalom and potential additional therapeutic factors as coding categories. The factors were related to outcome, operationalized as a decrease in psychological symptoms as measured with the Symptom Check List 90 (SCL-90).

**Results:**

All therapeutic factors of Yalom and four new factors were identified in the letters, ranging from 1 to 97%. The factors of ‘cohesion’ (97%), ‘interpersonal learning output’ (94%), ‘guidance’ (98%) and ‘identification’ (94%) were found in most letters. By contrast, ‘universality’ (1%), ‘family re-enactment’ (3%) and ‘instillation of hope’ (1%) were found in very few letters. The factors ‘interpersonal learning input’, ‘self-esteem’ and ‘turning point’ were significantly associated with therapeutic recovery.

**Conclusions:**

Large presence differences were encountered in therapeutic factors associated with resilience processes and the resolution of psychological distress. Although a relationship was found between certain factors and change in symptoms, it was unclear whether the factors had led to such change. Further research seems important for treatment in general and for the personalization of treatment.

## Background

Psychotherapeutic practices for youth show a great variety in treatment approaches deriving from different theoretical orientations [1, 2]. Although many adolescents benefit from psychotherapy, for others the outcome is discouraging [2, 3]. Against this background, it is understandable that there is a tendency to search for effective elements of mental care for youth [2, 4]. Therefore, examining the therapeutic factors related to successful treatment of adolescents may help therapists to optimize the treatment outcomes for this population, particularly for severely disordered groups such as young people with personality disorders. Mixed-method research with adolescents who report on the outcome of their individual treatment can help to provide an understanding of the success factors [5, 6]. Hence, the aim of this study was to identify such therapeutic factors in ego narratives written without instruction by a high-risk adolescent sample after treatment for a personality disorder, and to relate these to changes in symptoms during treatment.

Although as effective as individual therapy [7], it is argued that group psychotherapy, with its focus on peer relationships and identity formation, is preferable for adolescents [8]. To provide an understanding of clients’ perceptions of the effectiveness of group psychotherapy in general, Corsini and Rosenberg [9] and later on Yalom [10] devised the concept of therapeutic factors. The definitions of this concept vary, but typically the term refers to ‘curative factors’ or ‘mechanisms of change that occur through an intrinsic interplay of varied guided human experiences’ [11]. Yalom’s 12 therapeutic factors generated from his questionnaire were as follows: altruism, cohesion, universality, interpersonal learning input and output, guidance, catharsis, identification, family re-enactment, self-understanding, instillation of hope, and existential factors. They are now widely accepted as corresponding to relevant and potent mechanisms that bring about changes through group psychotherapy.

Yalom’s therapeutic factors in group psychotherapy have been studied in different group settings in a dozen studies, using Yalom’s group therapeutic factors questionnaire [11, 12]. However, until this study, no research had examined reports written by patients about therapeutic factors that contributed to their recovery. In addition, no researchers had focused on identifying therapeutic factors related to inpatient group treatment for adolescents with personality disorders or high-risk adolescents. In self-report studies on Yalom’s therapeutic factors, ‘cohesion’ is considered the central therapeutic factor that facilitates the other factors [13]. However, the interplay between all therapeutic factors, and the value placed on each, differs according to the content and purpose of a group [11]. One study on inpatient adolescent group therapy reported that ‘cohesion’, ‘universality’ and ‘instillation of hope’ were the most valued therapeutic factors [8]. Another study found that inpatients with comorbid personality disorder scored significantly higher on ‘family re-enactment’ and ‘self-understanding’ than patients without comorbid personality disorder, and significantly lower on ‘cohesiveness’ [14]. The investigation of unstructured reports of therapy outcomes, written by patients without instruction, might reveal other therapeutic factors or alter the rankings of importance among such factors.

In this mixed-method study, therapeutic factors related to patients’ reported recovery were examined for a high-risk adolescent population who had been clinically diagnosed with personality disorders. As part of a goodbye ritual at the end of an intensive group psychotherapy programme, participants were asked to write a farewell letter to express their thoughts and feelings about the treatment. This letter was read aloud to the group and treatment staff. Using content analysis [15], these farewell letters were studied to identify the therapeutic factors of Yalom [11]. Guiding questions were, first, which therapeutic factors were mentioned in the letters, and how often; second, which therapeutic factors could be related to a reduction in psychological stress and symptoms during treatment. Based on previous studies, it was expected that first, all of Yalom’s therapeutic factors would appear in the letters guided by the hypothesis that working in a group with peers using a group psychodynamic approach [11] would provide a positive influence; second, the therapeutic factors of ‘family re-enactment’ and ‘self-understanding’ were expected to be related to significant less psychological stress and symptoms at the end of the treatment following the study of Sayin [14] by subtracting the post-treatment total score on the SCL-90 from the pre-treatment score.

## Methods

### Participants

The participants were adolescents who had voluntarily been admitted to a partial residential mentalization-based treatment (MBT) facility of a youth psychiatry institution in the urban area of The Hague in The Netherlands. They had clinically diagnosed personality disorders and non-psychotic co-morbidity, and had completed the treatment according to protocol. Referrals came non-systematically from other mental health professionals, both within and outside the mental health care institution.

Between 2008 and 2017, 70 farewell letters were collected along with pre- and post-treatment data from the SCL-90. The adolescents’ mean age at the end of treatment was 18.9 years (*SD *= 1.7, range = 16–23) and most (88.6%) of the group were female. The average duration of treatment was a year, with a maximum of 18 months. Their intelligence, estimated from their level of education, was average to above average. Dutch was spoken fluently by all participants. In Table 1 an overview of the study population is given.

**Table 1:** Overview of study population on gender, DSM-IV Axis I classification and Axis II personality disorders (N = 70)

	n	%
Gender		
Female	62	88.6
Male	8	11.4
Axis I disorders		
Mood disorders	41	58.6
Anxiety disorders	22	31.4
Identity disorder	11	16.0
Eating disorders	9	12.9
Substance dependence	5	7.1
Dissociative disorders	2	2.8
Obsessive compulsive disorder	1	1.4
Attention deficit hyperactivity disorder	6	8.6
Axis II disorders		
No PD	6	8.6
One PD	57	35.7
Two PD’s	5	7.1
Three PD’s	1	1.4
Four PD’s	1	1.4
Paranoid PD	1	1.4
Antisocial PD	1	1.4
Borderline PD	18	25.7
Avoidant PD	16	22.9
Dependant PD	2	2.9
Obsessive compulsive PD	1	1.4
PD NOS	35	50.0

The excluded 32 patients with pre- and post-SCL-90 data but without a farewell letter, did not differ significantly from the others in age, gender, severity of symptoms, personality disorders, or duration of treatment from the rest of the sample

*PD* personality disorder

### Setting

The studied facility is named Albatros; it offers a 5-day-a-week structured and integrative psychodynamic group psychotherapy programme. Therapy often starts with residential treatment and then becomes day treatment during the treatment process. This intensive group psychotherapy is adapted for adolescents in an MBT programme [16–18] facilitated by a multidisciplinary team trained in MBT. The programme differs from the MBT programme offered for adolescents in England [19] using the psychodynamic group psychotherapy approach. The mentalizing focus of the various therapies is the adolescent’s subjective experience of himself or herself and others, and on relationships with group members and staff. Weekly verbal and non-verbal group psychotherapies, such as group psychotherapy, art therapy and psychodrama therapy are offered, combined with individual and family psychotherapy. Rituals form part of the programme—such as a birthday ritual, an old and new year’s ritual, and a farewell ritual. As the therapy programme progresses, each group member is given more responsibility regarding their participation in society and for other group members and group psychotherapy culture. If necessary, medication is prescribed according to protocol by a psychiatrist on the staff.

### Measures

Only participants who completed the treatment programme as planned wrote a farewell letter as part of a ritual at the end of treatment. At the start and end of treatment, the Symptom Check List 90 (SCL-90) [20] was completed.

#### Farewell letters

As part of the farewell ritual, the farewell letter is read to group members, treatment staff and one or two important persons outside of the treatment. No writing instruction was given, but the participants were familiar with the farewell letters of former group members. This familiarity meant that certain standard components appeared in most of the letters. All farewell letters were kept in folders accessible to the patients.

#### Scl-90

The authorized Dutch version of the SCL-90 [21] is a questionnaire with 90 questions; it uses a 5-point rating scale ranging from 1 (‘not at all’) to 5 (extreme response). The questionnaire assesses general psychological distress and specific primary psychological symptoms of distress during the last week. Outcome scores are divided into nine symptom subscales: anxiety, agoraphobia, depression, somatization, insufficient thinking and handling, distrust and interpersonal sensitivity, hostility, sleeping disorders, and rest. The total score (range 90–450) is calculated by adding the scores of the subscales. The test–retest reliability has been shown to be fair to good (*k* = .62–.91) [21].

### Procedures

During a 9-year period (2008–2017) all newly admitted adolescents were asked to participate in the study. A verbal description of the treatment protocol was provided to the participants. Then their written informed consent was obtained, according to legislation, namely the institution’s policy and Dutch law [22]. All patients agreed to participate, and in accordance with institutional policy they received no incentives or rewards. The procedures in this study were in accordance with the 1964 Helsinki declaration and its later amendments and comparable ethical standards. According to the treatment protocol, patients who finished treatment as planned were asked to write a farewell letter. The letter was read as part of the farewell ritual.

### Analysis

#### Content analysis

The first author and a senior colleague who is a psychologist were both part of the treatment team of the researched facility. They familiarized themselves with Yalom’s 12 therapeutic factors on the basis of Yalom’s 60-item group therapeutic-factor list [11]. The sample of 70 farewell letters was then examined using content analysis [23]. This qualitative method of analysis started with the first author reading ten letters while taking notes of themes, therapeutic factors of Yalom, and potential additional therapeutic factors. All sentences in the letters were numbered to compare the results of the coders. Thereafter, seven other farewell letters were coded independently by the two psychologists. All therapeutic factors found, which were not proposed by Yalom, were tracked systematically. The results were discussed regarding the use of the 12 factors and the identification of additional therapeutic factors. The maximum number of factors per sentence was limited to five.

Next, the remaining letters were analysed by the two psychologists, who coded every line for therapeutic factors based on Yalom’s 60-item group therapeutic-factor list and the additional therapeutic factors. The inter-rater reliability was determined by analysing which therapeutic factors occurred for which respondent, regardless of the number of times the factors occurred per respondent. The inter-rater reliability qualified as almost perfect (*k* = .83) [24].

The therapeutic factor ‘cohesion’ was most recognized by both psychologists, and ‘family re-enactment’ the least. Only factors about which the raters agreed were used; factors for which there was no agreement were not used in further analyses. Some therapeutic factors [2, 5, 8] were mentioned by almost every participant while others occurred almost never [3, 9, 11]. Because the aim of this study was to identify factors that differentiated between successful and unsuccessful treatments, factors that were not expected to differentiate because of low or high frequency were excluded from further analysis.

#### Statistical analysis

All quantitative analyses were performed using the Statistical Package for the Social Sciences, version 23.0 [25]. To operationalize therapeutic success, an SCL-90 outcome score was composed by subtracting the post-treatment total score from the total pre-treatment score. To compare the total score on the SCL-90 at the beginning of treatment with the end of treatment an ANOVA was used. Next, it was investigated which of the 12 plus four additional therapeutic factors correlated with this SCL-90 outcome score. Linear regression analysis was used to explore the relationship between the predictor variables (therapeutic factors) and the SCL-90 outcome scores.

## Results

### Results of content analysis

When comparing the pre- and post-treatment SCL-90 total data, a significant decrease in symptoms was found (*t *= 7.257, *p* = .000). The mean t-1 total score of 238.36 (*SD* = 50.93) on the SCL-90 declined to 186.86 (*SD* = 62.96) at t-2 (*d* = .90, 95% CI [37.34–65.66]). Content analysis of the 70 farewell letters showed that the patients generally summarised their struggles before treatment, followed by a description of the therapeutic process and the contact with group members and treatment staff. Most letters followed the same structure, starting with a salutation to the patient group and a description of how it feels to say goodbye; this was followed by a narrative of the participant’s mental state and struggle before or at the start of treatment, and a first impression of the patient group and group psychotherapy culture at the start of treatment. They described the psychotherapeutic interventions and contact with other patients, staff members, and loved ones. Many people mentioned the high points in the therapeutic programme, such as camping, practical jokes, and the changes they made, and ended by thanking and empowering the group members.

All 12 therapeutic factors of Yalom and four additional therapeutic factors—namely ‘self-esteem’, ‘turning point’, ‘resilience’ and ‘epistemic trust’—were identified. The final 16 therapeutic factors are described in detail below, with the use of illustrative quotations (noted by both psychologists) for their richness of description. To outline the context of an example, a quotation sometimes contains more sentences than the one that was associated with that specific factor. This example might also illustrate other therapeutic factors that were detected. Moreover, certain therapeutic factors seemed inevitably linked to each other. For example, the therapeutic factor ‘cohesion’ seemed to provide a necessary basis for ‘interpersonal learning’ factors; thus, these factors were often found together. The number of participants who mentioned specific therapeutic factors appears in the quantitative section. Indicators of frequency were employed to categorise quantity as follows: ‘many’ (approximately 85% or more), ‘most’ (more than 50%), ‘minority’ (less than 50%), and ‘few’ (less than 15%).

#### Altruism

‘Altruism’ was defined as group members helping and supporting one another. This factor was mentioned by a minority of participants, who encouraged the group to persevere and to believe in themselves. In addition, wise words, song lyrics and poems were included to illustrate this point.

*‘Fortunately, there were many people around me to help me.’*

#### Cohesion

‘Cohesion’ was defined as a sense of belonging to the group and being understood and accepted. This factor was expressed in many ways in the farewell letters, for instance by saluting the group with a nickname and using metaphors for the facility.

*‘Dear, dear, dear, dear, dear, dear everybody,*

*Yes, the little bird is ready to spread her wings and fly out of the nest. The nest that’s called the Albatros.’*

Almost all letters contained a paragraph describing in detail the joyful and playful moments among the group members, as well as shared moments of despair. Furthermore, participants expressed their gratitude to group members and treatment staff.

*‘Besides all the heavy therapies and tears, I have also experienced so much fun with you Albatrosses.’*

*‘What I also remember very strongly from my first week is the water and flour fight with group evening. In our wet and dirty clothes we walked back from a great fight to drink hot chocolate milk.’*

‘*Even though some periods were really difficult and sometimes I really wanted to go home, the nice, fun and cosy moments I will never forget, for example playing cards at night in the hallway, singing with a washing*-*up brush or just standing outside and chatting with everyone and many more things.’*

*‘Dear Albatrosses, I am going to miss you very much. I have experienced so many high and low points with you. I laughed and cried with you. You have all become special to me.’*

#### Universality

‘Universality’ was described as the importance of recognising one another, and the sense of not being the only person to feel a certain way. Remarkably few participants mentioned this factor.

‘*I want to thank the group for the recognition.’*

#### Interpersonal learning input

‘Interpersonal learning input’ was characterized as having learned how to present oneself to others. Most respondents referred to this factor. Receiving feedback was mentioned as valuable but difficult. Feedback from both group members and staff (which also counted as guidance) was described in the following quotes.

*‘My reactions to others were often unpredictable and caused a lot of insecurity in the group. An example was my suicide attempt in the beginning of my treatment. I have scared many groupmates with this and still regret it to this day.’*

*‘I was shocked, but accepted the tips. Eventually I started working on it, because yes, I really needed that kick in the pants.’*

*’It was difficult but due to the confrontations and support I received, I was able to take steps.’*

*‘Thank you for helping me to get to know myself. Thank you for having taught me that I am allowed to be vulnerable. Thank you for your commitment and patience.’*

#### Interpersonal learning output

‘Interpersonal learning output’ was defined as learning how to relate to others. Many participants described how they became familiar with group members and with other people.

*‘I notice that I learned the most of my groupmates because with you I have been able to practice with things that I found difficult, like appealing to people.’*

*‘It is now normal for me to talk in a large group about myself and to give my sometimes unpopular critical opinion.’*

*‘It was very safe and very familiar, and it was very nice to be able to sit at the table with fellow group members and team members, and talk nicely.’*

#### Guidance

‘Guidance’ was defined as group members receiving helpful, accurate information and therapeutic interventions. Many different therapies and therapeutic interventions were mentioned. First, the inpatient treatment itself was seen as an important step in breaking through fixed patterns, by being away from home and in a new environment.

*‘After my long crisis period of about 2.5* *months in the closed ward, I finally opted for treatment. I was terrified by this big step.’*

Second, specific interventions by the treatment team were mentioned as confrontational and difficult, but also as crucial for the process of change.

*‘After a while the care ban came. The (symbolic) care desk closed, and suddenly it was about me!’* (This therapeutic intervention was aimed at stopping the patient from focussing on and caring for others so that she could first take care of herself.)

Third, specific therapy forms were cited; these included individual and family group psychotherapies, such as EMDR and psychodrama therapy. Family therapy, specifically, was mentioned in the context of revealing family secrets or breaking through symbiotic relationships.

*‘And as if the feeling was not heavy enough, the team decided to speed up the process. I got the choice: whether you share your trauma with your parents or otherwise your treatment stops sooner.’*

Fourth, therapeutic alliances and contact with specific persons on the treatment staff were cited.

*‘I had damaged the trust of the group and the team, and had to think about what I had done and how I wanted to restore confidence again. In retrospect, I am very grateful for it, because this was really a turning point in my treatment.’*

Fifth, having to complete adolescent tasks, such as going to school, taking a job and practising hobbies, was described by many as not having been easy.

*‘I started school, oh dear that made me scared, I did not even dare to stand up and walk through the classroom. Luckily I started with a slow build*-*up programme.’*

#### Catharsis

‘Catharsis’ was characterized as the process of learning to cope with and to express painful emotions, and was described by many patients. Certain moments when they succeeded for the first time in being honest about their feelings and showing them, were described as important.

*‘I showed my sadness and anger. It was weight off* my *shoulders. It all became much calmer, not only in my head but also in my stomach.’*

Metaphors like wearing a mask were used to describe their old way of dealing with stress and negative emotions.

*‘I want to thank you for the fact that I was allowed to have my fighter jacket on, but especially for helping to take it off.’* (In this example, taking the fighter jacket off meant showing emotions in contact with the group instead of pushing the group away.)

#### Identification

Successful behaviour among group members was imitated by many; ‘identification’, in the sense that group members and team members provided examples for new behaviours, was not found.

*‘Hello dear group and team, first the well*-*known phrase: here I sit, on the farewell bench.* (This farewell bench referred to a seat on which the departing group member sat during the farewell ritual, and wrote his or her name. The sentence ‘Here I sit on the farewell bench’ occurred in almost every farewell letter).

#### Family re-enactment

‘Family re-enactment’ was defined as freeing group members from familial roles. A few patients mentioned being freed of their familial roles, or that being part of a group had helped them to relive and understand the family in which they had grown up.

*‘Some of you were just like little sisters for me. Due to that awareness and that experience with you, my relationship with my sister has become a lot better.’*

#### Self-understanding

‘Self-understanding’ referred to discovering and accepting previously unknown or unaccepted parts of oneself. Self-understanding was described by a minority.

*‘I know myself better now. I understand why I do what I do.’*

#### Instillation of hope

A few patients mentioned ‘instillation of hope’ by indicating that change was possible. Authors of such farewell letters encouraged other group members not to give up their hope for change. In addition, the ritual of reading a farewell letter itself had the goal of instillation of hope.

*‘I saw the Albatros as the last chance to make me feel better and finish my schooling.’*

#### Existential factors

A minority referred to ‘existential factors’, defined as taking responsibility for their lives while accepting the good and bad aspects. Also, participants mentioned having learned to accept negative emotions as part of life.

*‘I am willing to face the world, to have good but also bad times, and to take control of my own life again.’*

*‘I can only make myself happy, that is one of the things I have learned in my treatment on the Albatros.’*

*‘Feelings that unfortunately belong to me, which I dare to feel and accept.’*

#### Self-esteem

*‘*Self-esteem’ was described as a sense of being valued by the group and feeling self-confident. It was expressed by a minority. In this context, finding oneself was sometimes mentioned, as well as the sense of belonging.

*‘But not anymore; I am full of self*-*confidence, and I am myself.’*

*‘But above all that I am capable of much more than I think myself.’*

#### Turning-point

A few participants pointed out a crucial moment of change in their treatment. Some of these ‘turning points’ were due to therapeutic interventions or changes in the treatment programme.

*‘From the moment I went to day treatment, there was a turning point in my treatment for me.’*

*‘And when the subject was raised by Willy and Pieter, I found out that I was completely lost in caring for others. I therefore did not do anything about my own problems and felt incredibly depressed. That conversation with Willy and Pieter was a turning point for me in my treatment.’*

*‘I did a psychodrama about my acting out and how it got in the way of the contact with the group, and that really was the turning point for me.’*

Other turning points were due to the group and treatment staff demanding that a patient should try out new behaviour, or setting boundaries for behaviour that undermined change. Participants described receiving a supportive reassessment of treatment from a member of the treatment staff, in the presence of a group member as support, which was experienced as a ‘wakeup call’.

*‘I actually only had contact with them* (subgroup) *and I was missed on the group, I felt unseen and I damaged myself so that I was seen. That is why I got a treatment policy conversation, I personally see this as one of the turning points in my treatment.’*

*‘The realization came when I thought that no one liked me anymore, nobody shared secrets with me anymore, I had to talk about real things. It was the end of the world for me, I even wanted to resign. Then I fell, something broke. People were not there for me to hurt or bully me but to see me as a person. I have jumped, the contact I have with people now is real and the real contact is 10 times better than that secrets hassle.’*

#### Resilience

‘Resilience’ was defined as the belief that once could cope with stressful life events. A few adolescents mentioned the topic of resilience. They described how they had learned to adapt without falling back into acting-out behaviour.

*‘I still find this difficult, but I can cope with it now.’*

*‘I am aware of how I feel at a moment and how I can deal with it, without falling into acting out.’*

*‘I sometimes feel sad or lonely, the difference is that it no longer feels endless, I know how to deal with it and that I can accept it.’*

#### Epistemic trust

A few participants mentioned ‘epistemic trust’, defined as the ability to learn from and trust others. Epistemic trust differs from for instance the factor interpersonal learning input in the fact that this ability enables social learning in an ever changing social and cultural context and allows individuals to benefit from their social environment [26] and therefore seems a precondition for the other therapeutic factors. Experiences in the therapeutic milieu were described as being a corrective emotional experience.

*‘The Albatros was a safe house for me, a house where I could trust everyone, which at first seemed impossible.’*

*‘Thank you for what you have shown me. For the fact that thanks to your help, things have become bearable and that I have learned to feel what it is really like to care for people and to be able to rely on them.’*

### Results of the quantitative analysis

The 70 analysed letters consisted of 4669 sentences in total. Each letter had an average of 66.7 sentences, with the shortest letter containing 17 and the longest 171. The frequency of occurrence of the 12 therapeutic factors of Yalom and the four new therapeutic factors per participant are presented in Table 2.

**Table 2:** Definition and frequency of therapeutic factors (number and percentage of participants who named the relevant factor; N = 70)

Therapeutic factor	Definition	Prevalence	
		*n*	%
1. Altruism	Members help one another through giving of themselves to others	26	37.1
2. Cohesion	The sense of belonging to the group and being understood and accepted	68	97.1
3. Universality	The sense of not being the only one to feel this way	1	1.4
4. Interpersonal learning input	Refers to members learning how they come across to others	36	51.4
5. Interpersonal learning output	Refers to members learning how to relate to others	66	94.3
6. Guidance	Group members receiving helpful, accurate information and therapeutic interventions	62	88.6
7. Catharsis	The expression of feelings, both positive and negative	39	55.7
8. Identification	Members imitate successful behaviours modelled by other members or the treatment staff	66	94.3
9. Family re-enactment	Frees group members from familial roles	2	2.9
10. Self-understanding	Refers to members discovering and accepting previously unknown or unacceptable parts of themselves	13	18.6
11. Instilling hope	Refers to sense that change is possible	1	1.4
12. Existential factors	Members learn to take responsibility for the way they live their lives	21	30.0
13 Self-esteem	A sense of worth within the group and of being self-confident	19	27.1
14. Turning point	Member pointing out a crucial moment of change in the group therapy	7	10.0
15. Resilience	The belief that one can cope with stressful life events	11	15.7
16. Epistemic trust	Learning to trust and learn from other people	9	12.9

Among the 11 therapeutic factors left for analysis, a significant correlation was found between the SCL-90 score change and three therapeutic factors. These factors were ‘interpersonal learning input’ (*r* = .336, *p* = .004), ‘self-esteem’ (*r *= .241, *p* = .044) and ‘turning point’ (*r *= .324, *p* = .006). Multiple regression was then used to assess whether these three Yalom factors [4, 13, 14] accurately predicted the SCL-90 score change. Preliminary analyses were conducted to ensure there were no violations of the assumptions of normality, linearity, multicollinearity and homoscedasticity. All three therapeutic factors were entered together into the model. The total variance explained by the model was 22.4% (*F* (3, 66) = 6.35; *p* = .001). Each of the three factors made a unique and statistically significant contribution to the model. The strongest predictor was ‘interpersonal learning input’, which contributed 6.5% to the variance, followed by ‘self-esteem’ (5.8%) and ‘turning point’ (5.1%).

## Discussion

The aim of this mixed-method study was to investigate whether the therapeutic factors proposed by Yalom, with potential additional therapeutic factors, featured in letters written by recovering adolescents after completing an intensive group psychotherapeutic MBT. In addition, the relationships between these therapeutic factors and changes in symptom scores were explored. In 70 farewell letters written (without instruction) by a high-risk adolescent sample, all the therapeutic factors of Yalom [11] were identified in association with resilience processes and the resolution of psychological distress among the participants. Large differences were observed in the number of respondents who mentioned specific therapeutic factors. The factors of ‘cohesion’, ‘interpersonal learning output’, ‘guidance’ and ‘identification’ were almost always mentioned, and are therefore considered important among adolescents with personality pathology. These therapeutic factors seem to be a precondition for variables that were associated with therapeutic success. Therefore, although it would be premature to propose firm clinical implications based on these findings, the data indicate with great caution that it may be beneficial for clinicians to consider certain focus points in intensive group psychotherapy for adolescents with personality disorders. Clinicians could focus on the following issues, in addition to the common therapeutic factors: a) how the group members come across to one another, b) their sense of being valued by the group, and c) demanding that patients try out new behaviour, and setting boundaries to acting-out behaviour that undermines change. Replication is necessary to determine the generalizability of these results to other intensive MBT services for adolescents with personality pathology.

Similarly, the large differences in the number of respondents who mentioned a certain therapeutic factor could also be indicative of the individual needs and reflections on what helped during treatment. The study presented here provided insights into the way adolescents with clinically diagnosed personality disorders described their treatment and treatment outcome. The farewell letters highlighted for instance the importance of positive experiences with the group and treatment staff in addition to the treatment of psychopathology. The goal of the inpatient treatment is not only diminishing psychopathology, but also stimulating positive affects and experiences with others through therapeutic factors such as ‘cohesion’ and ‘interpersonal output’.

Following this, the question arises whether the interplay of all therapeutic factors and the value placed on them in general might differ not only according to the content and purpose of a group [11] but also among individual group members. In that case, treatment could focus not only on diminishing symptoms, yet also on optimizing the therapeutic factors that are most important to each individual. Furthermore, writing a farewell letter as part of the farewell ritual at the end of the treatment seemed to stimulate patients’ reflection on their therapeutic process. This can be important to highlight the result obtained through treatment.

The validity of using questionnaires with a high-risk adolescent group with varying mental states is questionable. In this study, change in symptom scores on the SCL-90 were used as indicator of therapeutic success. However, according to the treatment staff, all participants in the studied sample finished their treatment successfully. Patients who were not successful were offered a different farewell ritual (without writing a letter) and their data were not included in this study. Therefore, written reflections on the treatment process and progress during treatment could be more indicative of therapeutic recovery than a questionnaire score for these patients. This information could provide important input for treatment staff regarding how to optimize individual therapeutic factors. The importance of individual therapeutic factors could also differ across the phases of the psychotherapy process. For instance, the therapeutic factors of ‘cohesion’ and ‘interpersonal output’ could be especially important for some patients in the first phase of treatment, to help them learn to connect with others. ‘Guidance’ and ‘interpersonal input’ could be important in the second and third phase to work through interpersonal problems. Therefore, written reflections on the treatment process and patients’ progress could provide treatment staff with input on how to optimize the therapeutic factors for an individual in each treatment phase.

This study is unique in that rather than using a questionnaire that asked about every therapeutic factor of Yalom, as occurs in most studies, therapeutic factors were detected from the farewell letters. It is conceivable that those therapeutic factors that were barely evident in this study might have been more strongly observed through a questionnaire. For instance, ‘identification’, in the sense of participants mentioning that group members and team members had served as an example for new behaviour, was not mentioned. Four therapeutic factors were encountered in addition to those of Yalom, namely ‘self-esteem’, ‘turning point’, ‘resilience’ and ‘epistemic trust’. Two out of the three therapeutic factors that were related to the reduction of symptoms, namely’ self-esteem’ and ‘turning point’, were newly identified therapeutic factors. Whereas the groups studied by Yalom were mostly weekly outpatient groups, the facility studied in this research offered a 5-day intensive group psychotherapy programme with continuous availability of MBT-trained nursing staff. Therapeutic method and treatment staff likely influence therapeutic factors and factor rankings. However, it remains unclear whether differences in intensity, treatment staff availability, and patient groups were related to the new therapeutic factors. Nevertheless, it seems advisable for adolescent clinical practice to demand that patients try out new behaviours and to set boundaries to acting-out behaviour that undermines psychotherapy. Future research is needed to examine whether the new factors are indeed therapeutic factors.

Limitations of this study should be mentioned. First one may wonder if the identified therapeutic factors were implied as important for recovery in their therapeutic interventions by the treatment staff and copied by the writers. It seems likely that the treatment staff would provide role models regarding attitudes and rules of engagement. For example, some adolescents seemed to have used psychological language in their letters. The question here is whether the contents of those letters resembled the patient’s own reality, or rather reflected the desire to please the group and treatment staff. The second shortcoming of this study is the limited generalizability of the results due to the use of inpatients at a single facility, and the small sample. Despite these limitations, the study remains valuable because little prior research had been done regarding personality disorders among adolescents [27–29].
